# Identifying Seasonal and Diurnal Variations and the Most Frequently Impacted Zone of Aerosols in the Aral Sea Region

**DOI:** 10.3390/ijerph192114144

**Published:** 2022-10-29

**Authors:** Yongxiao Ge, Na Wu, Jilili Abuduwaili, Rashid Kulmatov, Gulnura Issanova, Galymzhan Saparov

**Affiliations:** 1 State Key Laboratory of Desert and Oasis Ecology, Xinjiang Institute of Ecology and Geography, Chinese Academy of Sciences, Urumqi 830011, China; 2 CAS Research Center for Ecology and Environment of Central Asia, Urumqi 830011, China; 3 University of Chinese Academy of Sciences, Beijing 100049, China; 4 Department of Biology, National University of Uzbekistan, Tashkent 100170, Uzbekistan; 5 Kazakh Research Institute of Soil Science and Agrochemistry Named after U.U.Uspanov, Almaty 050060, Kazakhstan; 6 Faculty of Geography and Environmental Sciences, Al-Farabi Kazakh National University, Almaty 050040, Kazakhstan

**Keywords:** dust, terminal lake, aerosols, Aral Sea, Central Asia

## Abstract

With the desiccation of the Aral Sea, salt–alkali dust storms have increased in frequency and the surrounding environment has deteriorated. In order to increase our understanding of the characteristics and potential impact zone of atmospheric aerosols in the Aral Sea region, we evaluated seasonal and diurnal variation of aerosols and identified the zone most frequently impacted by aerosols from the Aral Sea region using CALIPSO data and the HYSPLIT model. The results showed that polluted dust and dust were the two most commonly observed aerosol subtypes in the Aral Sea region with the two accounting for over 75% of observed aerosols. Occurrence frequencies of polluted dust, clean continental, polluted continental/smoke, and elevated smoke showed obvious seasonal and diurnal variations, while occurrence frequency of dust only showed obvious seasonal variation. Vertically, the occurrence frequencies of all aerosol subtypes except dust showed significant diurnal variation at all levels. The thickness of polluted dust layers and dust layers exhibited same seasonal and diurnal variations with a value of more than 1.0 km year-round, and the layer thickness of clean continental and polluted continental/smoke shared the same seasonal and diurnal variation features. The zone most severely impacted by aerosols from the Aral Sea region, covering an area of approximately 2 million km^2^, was mainly distributed in the vicinity of the Aral Sea region, including western Kazakhstan, and most of Uzbekistan and Turkmenistan. The results provide direct support for positioning monitoring of aeolian dust deposition and human health protection in the Aral Sea region.

## 1. Introduction

Lakes represent important ecosystems that provide invaluable ecosystem services in terms of biodiversity, climate regulation, and tourism activities [1,2]. However, at present, anthropogenic influences on lakes are practically ubiquitous and lake ecosystems have suffered great losses from intensifying human activities and climate change [3,4,5,6]. Currently, concerns about biodiversity losses, ecological and environmental consequences, and hazards to human health have inspired the protracted use, better management, and more sustainable conservation of lakes worldwide.

Lakes in arid areas are at higher risk from human activities and climate change than those in wetter climates due to drought, strong evaporation, and stresses from agricultural irrigation and industry [7,8]. Consequently, many terminal lakes in arid central Asia have shrunk in recent decades due to intensifying human activities and climate change. The Lop Nor, located near the eastern edge of the Tarim Basin, has permanently dried out due to climate change and human activities, which is called the earliest Aral-Sea-type disaster in the world [9,10,11,12]. Due to damming and irrigation, the area of the Aral Sea has shrunk by 60,156.50 km^2^ (approximately 87%) over the period from 1960 to 2018 [13,14]. Since 2003, the area of Ebinur Lake in northwest China has declined dramatically from 817.63 km^2^ in 2003 to 384.60 km^2^ in 2015 [15]. In Iran, increased temperature, decreased rainfall, and mismanagement of water resources resulted in sharply declining water levels in the Urmia Lake basin from 1986 to 2017 [16,17]. In roughly two years, the Hamun Lakes on the Iran–Afghanistan border lost 89% of surface area as a result of the rapid expansion of irrigated agricultural lands and desiccated for the first time in August 2012 [18]. Balkhash Lake [19], Manas Lake [20], and Siletiteniz Lake [21] exhibit a tendency toward progressive desiccation and fluctuation of water levels, which result in land degradation and loss of ecosystem services in respect of wetlands. 

A significant number of aerosols, which can significantly affect the regional dynamics of the atmosphere and the climate system, can enter the atmosphere as a result of dust storms [22]. The desiccation of lakes in arid Central Asia has significantly deteriorated the local communities’ health and environmental conditions and led to high levels of atmospheric pollution of adjacent territories [23,24], involving loss of livelihoods, sand and dust storms, regional climate change, loss of important species, and in particular, many major health issues due to airborne salt–dust and dust. The exposed dry beds of shrinking lakes (namely, “playa”) play an important role in salt dust generation and have become a new active source of dust and anthropogenic pollutants [25,26,27]. The wind erosion of the saline playa of Ebinur Lake causes frequent salt–alkali dust storms, which seriously influence the normal absorption of minerals by plant leaves and impose risks on human health in this region [28,29]. The decline in the area of Urmia Lake has resulted in air pollution caused by PM_10_ (particulate matter with particle sizes below 10 microns), which is blown from the dry lakebed to the neighboring cities [30,31]. With the sharp decline in the water level of the Aral Sea, the surrounding environment of the Aral Sea has deteriorated rapidly. The rich and diverse ecosystems of the Amu Darya Delta in Uzbekistan and the Syr Darya Delta in Kazakhstan have both suffered substantial harm [32]. For example, the fauna and flora in the Aral Sea region have changed dramatically as a result of a significant increase in salinity [33]. Exposed lake playas have become a source of fine dust that is taken up during dust storms and distributed over great distances, damaging people’s health and the ecosystem in nearby areas [34,35]. At present, the new Aralkum Desert in the west part of the dry lakebed commonly experiences dust storm events at the highest frequencies in the world [36]. Suspended particles in the atmosphere cause anemia, diarrheal illnesses, and significant toxic pollutant burdens in the body, as well as respiratory diseases; the millions of people living in this region are suffering not only from natural disasters but also from a sharp decline in their health status [37,38,39,40], especially in Karakalpakstan in Uzbekistan and Kyzylorda in Kazakhstan. The monthly average dust deposition within the severely impacted zone is 598.4 kg/ha; during dust storm events, peak deposition is up to 160 times higher than the monthly average [41]. After being deposited on the ground by means of dry deposition or wet deposition, salt dust and dust will first have a great impact on the growth of crops such as cotton and wheat within the Amu Darya and Syr Darya basins by reducing the efficiency of photosynthesis in leaves and increasing the soil salt content, causing soil secondary salinization due to the higher salt content of the deposited dust [42,43]. The westerly driven potential long-range transportation of fine salt dust from the Aral Sea region will accelerate the melting of snow and ice by increasing the surface temperature due to the change in the albedo of the ice and snow in the Tianshan Mountains region [44,45], which will shorten the length of the snowmelt period, causing sudden floods that threaten the safety of humans and property in this region.

The Aral Sea catastrophe has been called the largest human-made ecological disaster in the 21st century and has been studied extensively by researchers, organizations, and policymakers all over the world [46,47]. Thus far, studies on the Aral Sea have involved water levels, environmental change, wind erosion, and ecological and environmental effects through research methods including field monitoring [41,48], model simulations [49,50,51], remote sensing [52,53], etc. The Aral Sea’s fluctuating water level and underlying surface have become more frequently discussed as a result of human activity and climate change [54,55,56]. For example, the Aral Sea’s variations in area and water volume between 1960 and 2018 were reconstructed by Yang et al. [14], and Huang et al. [57] explored the factors that have caused the Aral Sea to decline, from a combined perspective of climate, land, water, and ecological change. Many scholars have carried out studies on the entire wind erosion process, including dust emission, transport, and deposition, using field monitoring, model simulation, and remote sensing inversion [58,59,60,61]. Among the many studies, Karami et al. [49] analyzed two distinctive dust storm episodes from the Aral Sea region using three models. Wang et al. [62] investigated the temporal characterization of sand and dust storms and their possible driving factors in the Aral Sea region. Wu et al. [63] revealed the night-time variation features of aerosols from the Aral Sea region. However, the seasonal and diurnal variations of aerosols in the Aral Sea region are still unknown, and these variations generally determine their impact on health and climate in this region. The potential impact of pesticides, PM_10_ and PM_2.5_, and heavy metals carried by dust from the Aral Sea region on human health, ecology, and the environment has also been studied qualitatively [64,65]. Up to now, data in terms of the zone most frequently impacted by aerosols from the Aral Sea region have been scarce and limited. Despite previous efforts to analyze the Aral Sea Basin’s aerosol fluctuations and probable long-distance transport [61,63], a lack of seasonal and diurnal variations and the specific most frequently impacted zone in respect of aerosols in the Aral Sea region still exists. In the context of climate change, a more comprehensive and in-depth understanding of the variations and zone most frequently impacted by aerosols from the Aral Sea region is of great practical and scientific significance for taking effective measures to mitigate and deal with environmental and health disasters. 

In light of the present demand for a deeper comprehension of the variations and impact zone of aerosols from the Aral Sea region, in this study, we first aimed to evaluate and identify the day–night variations and then identify the most frequently impacted zone of aerosols through aerosol data derived from the Cloud–Aerosol Lidar and Infrared Pathfinder Satellite Observations (CALIPSO) satellite and the frequent analysis contained in the Hybrid Single Particle Lagrangian Integrated Trajectory (HYSPLIT) model.In this paper, we first shed light on seasonal and diurnal fluctuations in respect of subtypes, altitude profile, and thickness of aerosol subtype layers in the Aral Sea region and then identify the zone most frequently impacted by aerosols from this region. We do so in Section 3.1 by identifying the dominant aerosol types over the Aral Sea region. Our results in Section 3.2 present the seasonal and diurnal variation characteristics of different aerosol subtypes. Section 3.3 and Section 3.4 discuss seasonal and diurnal variations in the altitudes and thicknesses of the aerosol layers in detail. Finally, the most frequently impacted zone is identified in Section 3.5. The results of this paper will provide direct support for human health protection and positioning monitoring of aeolian dust deposition, and will also provide an impetus for the prudent use and comprehensive protection of the Aral Sea.

## 2. Regional Environment and Methodology

### 2.1. Regional Environments

**Figure 1:**
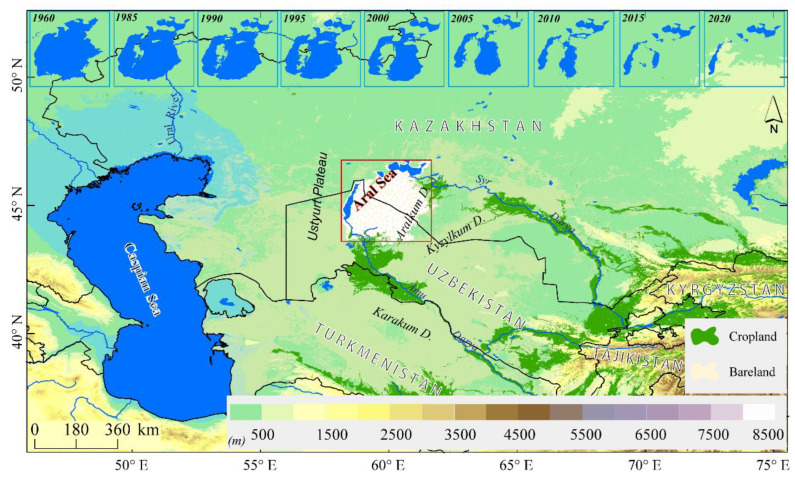
Geographic location of the Aral Sea in arid Central Asia and the change in area of the Aral Sea from 1960 to 2020. The annual area of the Aral Sea was derived from Landsat data; the cropland and bareland were extracted from the Global ESA CCI land cover classification map in 2020, with a spatial resolution of 300 m.

Central Asia has a continental arid climate and contains the world’s largest temperate desert ecosystem, comprising around a third of the world’s dryland area [66]. Its geographical location and arid climate make the region’s ecological environment very fragile [67]. Climate change and human disturbances can easily induce significant changes in the ecosystem or even cause severe ecological disasters due to the fragility of the ecosystem [68]. The gradual diminishment of the Aral Sea has evolved into a world-famous cross-national ecological disaster in arid Central Asia. The Aral Sea, formerly the world’s fourth-largest inland water body, is a vast terminal lake situated in Central Asia’s deserts [69]. Over the past 10 millennia, it has filled and dried numerous times due to both natural and human influences. The most recent desiccation event occurred in the early 1960s, when the area was around 6.75 × 10^4^ km^2^, and this was largely caused by increased irrigation water from the Amu Darya River and Syr Darya River, which finally feed into the Aral Sea. Under the influence of human activities, the South Aral Sea was separated into eastern and western sections in 2003 (Figure 1). In 2006, the Aral Sea was divided into three small lakes, covering an area of 1.7 × 10^4^ km^2^ [70], and by September 2009, the Aral Sea had separated into four distinct water bodies [71]. The Aral Sea’s ongoing declining tendency has not been halted. In 2021, it had a surface area of only 0.56 × 10^4^ km^2^, leaving 6.2 × 10^4^ km^2^ of playa around the present Aral Sea [63]. The eco-environment around the Aral Sea has deteriorated quickly, and a vast dry lakebed came into being and has turned into a source of fine salt-dust and dust due to strong wind erosion in the Aral Sea region. The dry lakebed of the Aral Sea and adjacent desert territory have become one of the world’s major sources of salt dust, and the Aral Sea’s surrounding population is suffering from serious health problems [69,72]. 

### 2.2. Methodology

#### 2.2.1. Variations in Aerosol Subtypes in the Aral Sea Region

The Cloud–Aerosol Lidar and Infrared Pathfinder Satellite Observations (CALIPSO) satellite is an Earth-observing satellite that was launched on 28 April 2006. The main instruments aboard CALIPSO include the Imaging Infrared Radiometer (IIR), the Cloud–Aerosol Lidar with Orthogonal Polarization (CALIOP), and the Wide Field Camera (WFC). CALIPSO is now the only satellite that can report the vertical distributions of aerosol spatial and optical properties during the daytime and nighttime over the whole world [73], which makes it possible for us to clarify the seasonal and diurnal variations of aerosol subtypes. 

There are three levels of CALIPSO data available. Level 1 products provide raw data with high spatial resolution, Level 2 products classify aerosol subtypes through a scene classification algorithm, and specific monthly variables derived from Level 2 items are provided by Level 3 products. The Level 2 CALIPSO data can be classified into three types: the cloud and aerosol layer data product, the cloud and aerosol vertical profile data product, and the vertical feature mask data product [74]. In the present study, all accessible level 2 version 4 CALIPSO layer products in respect of the Aral Sea region (Figure 1) for the period from 2007 to 2021were analyzed. 

There are seven aerosol subtypes for tropospheric aerosols (dust, polluted dust, clean continental, polluted continental/smoke, elevated smoke, marine, and dusty marine) and four aerosol subtypes for stratospheric aerosols (polar stratospheric aerosol (PSA), volcanic ash, sulfate/other, and smoke) when using a scene classification algorithm [73]. Following Han et al. [75] and Wu et al. [63], aerosol subtypes with an absolute CAD score (cloud–aerosol discrimination) ≥70 were selected to expound day-night variation in atmospheric aerosols in the Aral Sea region in spring (MAM, from March to May), summer (JJA, from June to August), autumn (SON, from September to November), and winter (DJF, from December to February).

#### 2.2.2. Identification of Most Frequently Impacted Zone 

The Hybrid Single-Particle Lagrangian Integrated Trajectory model (HYSPLIT), developed by the Air Resources Laboratory of the NOAA, has been utilized in modeling the transport, dispersion, and deposition of pollutants and hazardous compounds in a number of simulations [76,77]. In the present study, the HYSPLIT model, driven by Global Data Assimilation System (GDAS) data from 2007 to 2021, was first used to obtain the hourly forward air parcel trajectory for a 72 h period centered on the Aral Sea from 2007 to 2021. Then, the HYSPLIT model was used to identify the zone most frequently impacted by atmospheric aerosols from the Aral Sea region in spring, summer, autumn, and winter through the air-parcel trajectory frequency analysis method contained in the model. The technical instructions on the NOAA website go into great depth regarding the trajectory frequency analysis.

## 3. Results and Discussion

### 3.1. Diurnal Variations in the Percentage of Aerosol Subtypes over the Past Fifteen Years in the Aral Sea Region

Atmospheric aerosols are typically a combination of several subtypes rather than a single type. The total number of CALIPSO aerosol subtype measurements in the daytime (21,040) and nighttime (65,964) showed significant differences (Table 1). In general, due to the stronger SNR (signal-to-noise ratio), CALIPSO finds more atmospheric aerosol subtype samples in the nighttime than in the daytime [78,79]. In this study, the percentage of each aerosol subtype was calculated by dividing the number of one aerosol subtype detected by the total number of CALIPSO aerosol subtype measurements. Table 1 presents the percentage differences between aerosol subtypes in the daytime and nighttime from 2007 to 2020. The percentages of dust were 58% and 25% in the daytime and nighttime, respectively. It should be noted that the percentage of dust aerosols in the daytime means a higher percentage as a fraction of all the CALIPSO aerosol subtypes observed in the daytime over the past fifteen years, but that does not necessarily mean that more dust layers were detected. The same applies to the percentage differences in other aerosol subtypes. Polluted dust accounted for 27% and 50% of all aerosols detected in the daytime and nighttime, respectively (Table 1). The percentage of polluted continental/smoke was 9% in the daytime and nighttime, which is particularly remarkable among the five aerosol subtypes, but that does not mean that the same amounts of polluted continental/smoke layers were detected. The minimum percentage (2%) of aerosol subtypes was observed in the clean continental subtype in the daytime, while in the nighttime, the percentage was 9%—the percentage was much higher than that in the daytime when taking the total number of CALIPSO aerosol subtype measurements into consideration. Elevated smoke had a percentage of 3% in the daytime, and the percentage in the nighttime was twice as much as that during the daytime. Dust and polluted dust were the two dominant atmospheric aerosol subtypes over the Aral Sea region (Table 1), which is consistent with the findings of Wu et al. [63]. The dust and polluted dust were the two most commonly observed aerosol subtypes for both nighttime and daytime, with the two accounting for over 75% of observed aerosols. 

**Figure 2:**
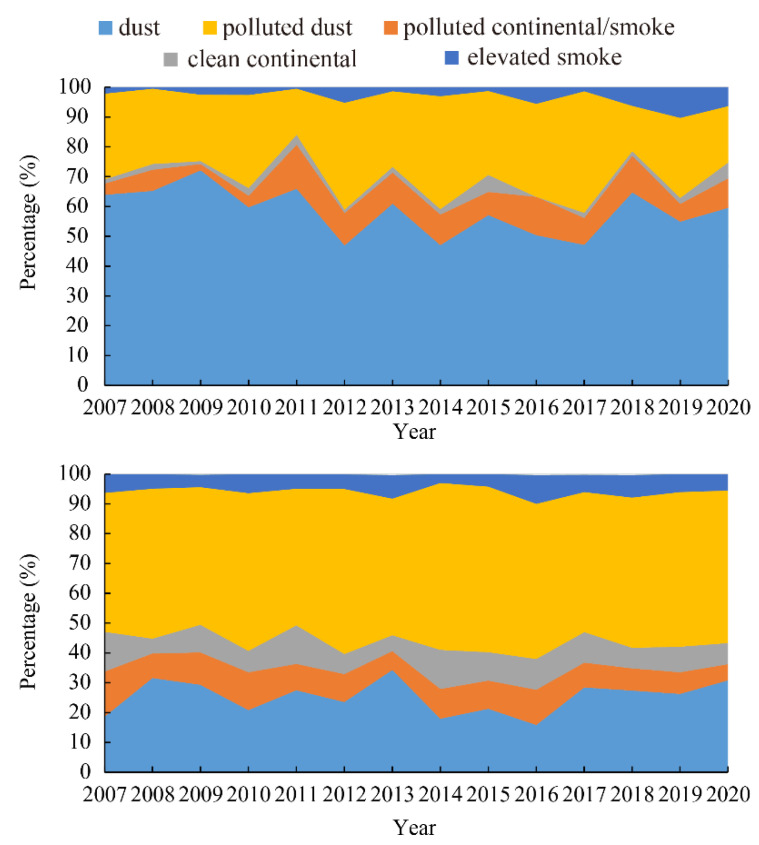
Change in percentage of aerosol subtypes in the daytime (the **top** panel) and nighttime (the **bottom** panel) from 2007 to 2020 in the Aral Sea region.

The percentages of aerosol subtypes in the daytime from 2007 to 2020 are depicted in the top panel of Figure 2. Dust and polluted dust were the two dominant atmospheric aerosols over the past fifteen years (Figure 2), which is in line with the results above. The percentage of dust varied between 47% and 72%, showing a fluctuation of around 58%. The percentage of polluted dust varied from 15% to 41% with a mean value of 27%. That of polluted continental/smoke did not show a significant difference and was around 9% from 2007 to 2020. Clean continental showed almost the same percentage wtihin the research period, with a mean value of approximately 3%. The obvious difference is that elevated smoke exhibited a slight increasing trend in percentage, with the value changing from 1% to 10%.

The percentage of aerosol subtypes for the nighttime from 2007 to 2020 is depicted in the bottom panel of Figure 2. Dust and polluted dust were still the two dominant atmospheric aerosol subtypes; however, polluted dust became the dominant aerosol subtype, the percentage of which varied between 46% and 56%, showing a slightly increasing trend. The percentage of dust varied from 16% to 32%, with a mean value of 25%; there was little difference in percentage between polluted continental/smoke and clean continental with an average percentage of approximately 9%. The elevated smoke showed a stable trend with a mean value of 6%. 

In the present study, the percentage of dust subtype detected during the daytime was almost 2 times larger than polluted dust; however, conversely, the percentage of polluted dust in the nighttime was two times larger than dust over the past fifteen years. The change in the percentage of aerosol subtypes in the daytime (the top panel in Figure 2) and nighttime (the bottom panel in Figure 2) from 2007 to 2020 over the Aral Sea also showed the same feature. This may be due to three factors. Firstly, sunlight in the daytime increases the noise, which will impact the lidar signal, reducing the polluted dust layer detection sensitivity. Secondly, in general, the dust layers are thicker than the polluted dust [63]. Thirdly, polluted dust, which contains scattering aerosols (such as sulfate and nitrate aerosols), impacts the reflected signal significantly in the daytime. A large part of the presented difference between dust and polluted dust in the nighttime is attributed to the ability of the lidar to detect more tenuous aerosol layers in the nighttime [80]. 

### 3.2. Seasonal and Diurnal Variations in Occurrence Frequency of Aerosol Subtypes in the Aral Sea Region

**Figure 3:**
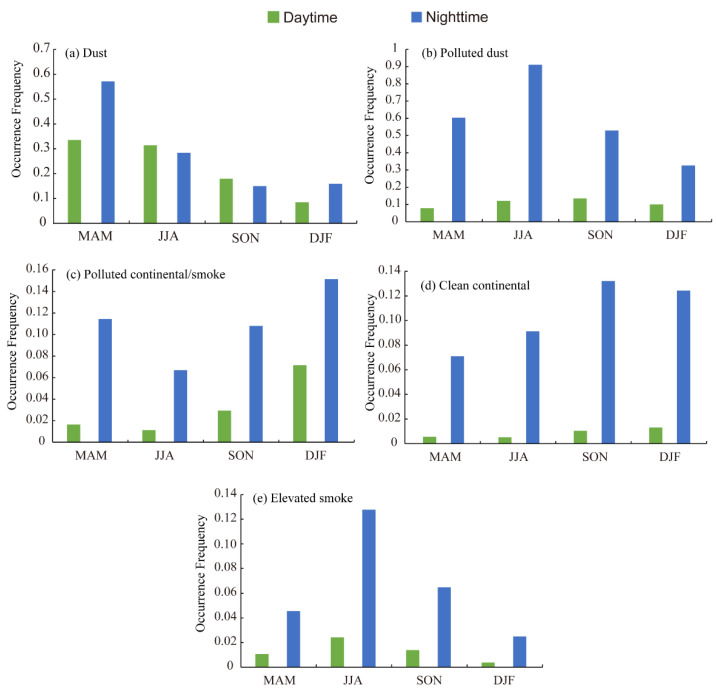
Seasonal and diurnal variations in occurrence frequency of aerosol subtypes in the daytime and nighttime from 2007 to 2021: (**a**) dust; (**b**) polluted dust; (**c**) clean continental; (**d**) polluted continental/smoke; (**e**) elevated smoke.

The seasonal and diurnal variations in the occurrence frequency of aerosol subtypes in the Aral Sea region are shown in Figure 3. Following Wu et al. [63], the occurrence frequency was derived from CALIPSO data through the number of CALIPSO profiles obtaining aerosol subtypes retrieval divided by the total number of profiles within the research grid. Overall, the occurrence frequency of aerosols in the Aral Sea region observed by CALIPSO presented significant seasonal features (Figure 3). The occurrence frequency of dust in the daytime showed a decreasing trend from spring to winter, which peaked in spring, followed by 0.31 in summer, 0.18 in autumn, and 0.08 in winter. The occurrence frequency of dust in the nighttime showed the same trend, decreasing in order of spring (0.57), summer (0.28), autumn (0.15), and winter (0.16). There was no discernible variation in the occurrence frequency between the daytime and nighttime, except in spring, when the difference peaked (Figure 3a). The occurrence frequency of polluted dust in the daytime and nighttime showed significant differences, peaking in summer (Figure 3b). The seasonal features of polluted dust showed a unimodal distribution. In the daytime, the maximum occurrence frequency appeared in autumn (0.14), then decreased in the order of summer (0.12), winter (0.10), and spring (0.08); however, during the nighttime, the occurrence frequency peaked in summer (0.91). The well-captured seasonality of the dust and polluted dust occurrence frequencies reflects the maximum seasonal characteristics of dust activities in the Aral Sea region, which presented the same features as those of the Gobi, Taklimakan Desert, and Thar Desert [81] in arid central Asia. The occurrence frequency of elevated smoke showed the same trend as polluted dust (a significant difference existed between daytime and nighttime, which peaked in summer) and elevated smoke had a higher occurrence frequency in summer with values of 0.02 and 0.13 for the daytime and nighttime, respectively (Figure 3e). Polluted continental/smoke and clean continental occurrence frequencies increased in a comparable manner from spring to winter, with a considerable difference in occurrence frequency detected in the daytime and nighttime; namely, the occurrence frequency in the nighttime was much higher than that in the daytime.

**Figure 4:**
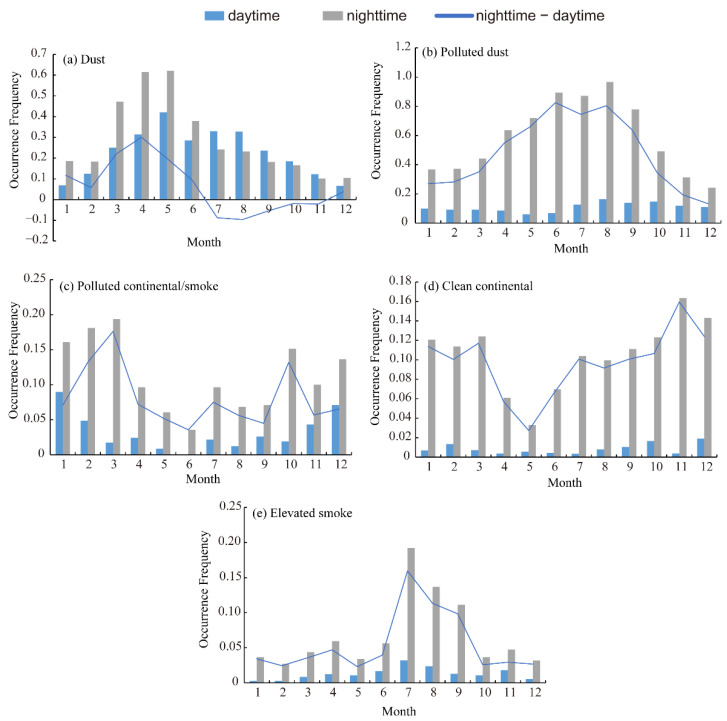
Monthly occurrence frequencies of five subtypes in the daytime and nighttime from 2007 to 2021: (**a**) dust; (**b**) polluted dust; (**c**) clean continental; (**d**) polluted continental/smoke; (**e**) elevated smoke.

Figure 4 depicts the monthly occurrence frequency of aerosol subtypes in the daytime and nighttime to better recognize the variation features of aerosol subtypes in the Aral Sea region, which shows a more marked seasonal and diurnal feature. In the daytime, the occurrence frequency of dust exhibited two distinct peaks in spring (May) and summer (July) with occurrence frequencies of 0.42 and 0.33, respectively. Meanwhile, the occurrence frequency in the nighttime showed only one peak in spring (May), with a value of 0.62, which was much higher than that in the daytime (Figure 4a). A significant difference in the occurrence frequency of dust was only presented from January to May, and the maximum difference appeared in April. After June, the difference between the daytime and nighttime was not as obvious. The occurrence frequencies of polluted dust, clean continental, elevated smoke, and polluted continental/smoke had large positive diurnal differences year-round (the occurrence frequency in the daytime was much higher than that found in the nighttime); this was different from what we discovered regarding the occurrence frequency of dust. The occurrence frequency of polluted dust in the daytime and nighttime exhibited the same unimodal distribution. The maximum occurrence frequency appeared in August, with values of 0.16 and 0.97 in the daytime and nighttime, respectively (Figure 4b). The difference also peaked in summer (maximum in June). The occurrence frequency of polluted continental/smoke presented the same trend as clean continental, with the minimum occurrence frequencies appearing in spring (May) and summer (June) and the maximum occurrence frequency appearing in winter, especially from November to February. The occurrence frequency of elevated smoke was lower than 0.2 year-round, exhibiting a unimodal distribution that peaked in summer (July), with values of 0.03 and 0.20 during the daytime and nighttime, respectively. 

It is notable that the diurnal variability of the dust occurrence frequency showed a much smaller difference compared with other subtypes detected in the Aral Sea region, which can be attributed to the ability of the lidar to detect the thicker dust layer, leading to insignificant differences in occurrence frequency in the daytime or nighttime. Therefore, observations for the daytime and nighttime can both reflect the variation in dust aerosols in the Aral Sea region (Figure 3 and Figure 4). The occurrence frequencies of the other aerosol subtypes (polluted dust, clean continental, polluted continental/smoke, and elevated smoke) showed larger diurnal variations compared with that of dust. Accordingly, observations at nighttime were the better choice by which to clarify the variation features of polluted dust (sulfates, nitrates), clean continental, polluted continental/smoke, and elevated smoke in the Aral Sea region. Another notable feature that needed to be taken into account in the present study is the seasonal fluctuation in the occurrence frequencies of dust and polluted dust. That is, the occurrence frequency of dust peaked in spring whereas the occurrence frequency of polluted dust peaked in summer. This can be attributed to the change in the moisture content and physical surface characteristics of the playa in the Aral Sea region. Polluted dust comprises dust minerals mixed with anthropogenic pollutants such as salt and heavy metals [28]. In spring, the highly moist surface and biological soil crusts improve the resistance of the playa to wind erosion [82], which leads to a lower occurrence frequency of polluted dust in spring compared with that in summer. However, the dust was mainly emitted from hyper-arid deserts such as the Aralkum Desert in the Aral Sea region, which were less affected by moisture and surface characteristics. Thus, the occurrence frequency of dust in the Aral Sea region peaked in spring, which is consistent with the conclusion drawn by Indoitu et al. [83], Zhang et al. [84], and Shi et al. [85]. In summer, the groundwater level of the Aral Sea dropped due to human activities and evaporation, leaving a salt desert landscape with a friable surface within the playa, which is highly susceptible to wind erosion and become the dominant source of polluted dust [86]. Therefore, the occurrence frequency of polluted dust was substantially higher than that in spring. Clean continental (also referred to as background aerosol) is a lightly loaded aerosol consisting of sulfates, nitrates, and ammonium, while polluted continental/smoke is background aerosol with a substantial fraction of urban pollution and/or smoke. The polluted continental/smoke and clean continental have the maximum occurrence frequency appearing in winter, which can be attributed to the high anthropogenic fossil fuel/biofuel consumption in Central Asian countries [87,88]. The elevated smoke, consisting primarily of soot and organic carbon [73], with occurrence frequency peaking in July, is primarily from biomass burning and fires in summer in arid central Asia [89,90].

### 3.3. Seasonal and Diurnal Variations in Vertical Distribution of Aerosol Subtype Layers in the Aral Sea Region 

**Figure 5:**
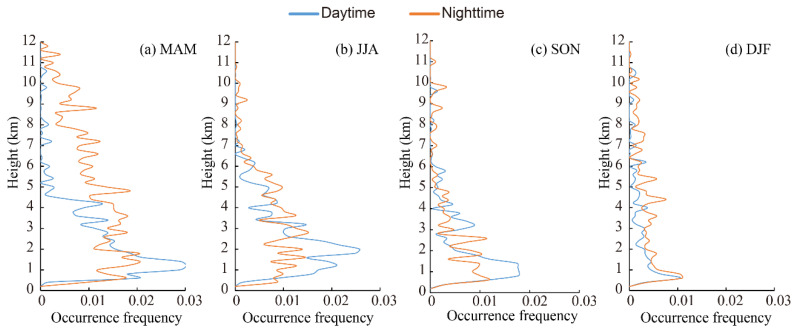
Vertical distribution of occurrence frequencies of dust layer in the daytime and nighttime in the Aral Sea region from CALIPSO observations for four seasons.

**Figure 6:**
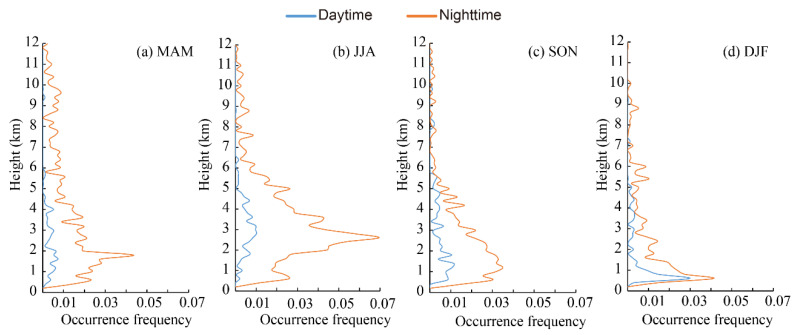
Vertical distribution of occurrence frequencies of polluted dust layer in the daytime and nighttime in the Aral Sea region from CALIPSO observations for four seasons.

**Figure 7:**
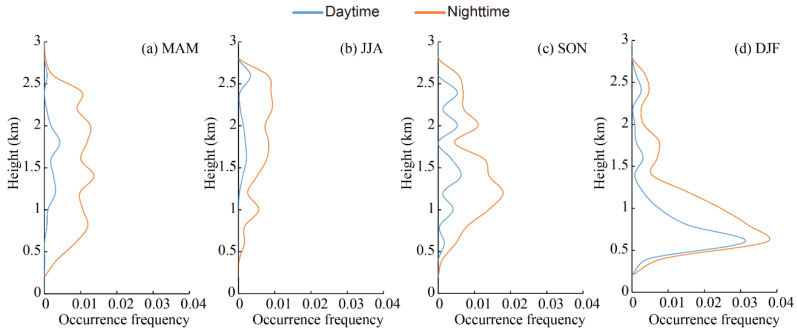
Vertical distribution of occurrence frequencies of polluted continental/smoke layer in the daytime and nighttime in the Aral Sea region from CALIPSO observations for four seasons.

**Figure 8:**
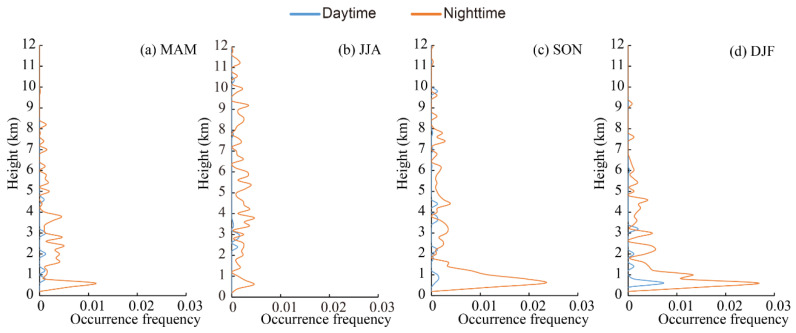
Vertical distribution of occurrence frequencies of clean continental layer in the daytime and nighttime in the Aral Sea region from CALIPSO observations for four seasons.

**Figure 9:**
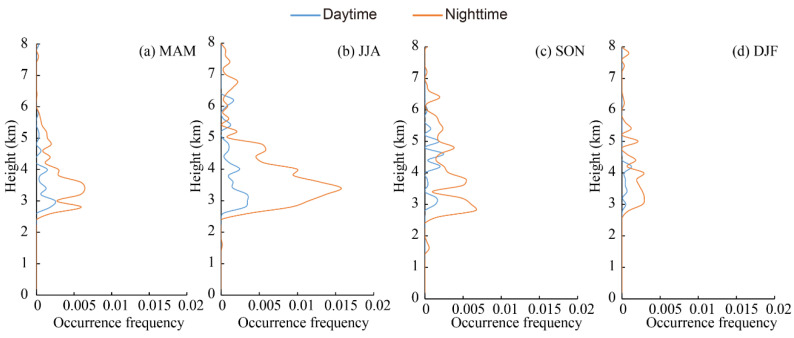
Vertical distribution of occurrence frequencies of elevated smoke layer in the daytime and nighttime in the Aral Sea region from CALIPSO observations for four seasons.

In general, the vertical distribution of aerosols dictates their health and climate implications [91]. The vertical profiles of the occurrence frequencies of the aerosol subtype layers observed in the daytime and nighttime over the Aral Sea region in different seasons are shown in Figure 5, Figure 6, Figure 7, Figure 8 and Figure 9. The altitudes of subtype layers were divided into different height intervals with 200 m bins. The vertical-resolved occurrence frequency of aerosol subtypes was calculated by dividing the number of aerosol subtypes detected at each given altitude bin by the total number of profiles. Dust highly overlapped and was distributed over wide ranges (Figure 5). Dust was more frequently observed at lower altitudes, especially between 0.5 km and 4.3 km above mean sea level, peaking at approximately 1–2 km and then decreasing as the height increased. The occurrence frequency of dust layers in the daytime and nighttime did not show significant diurnal differences over the four seasons, except for dust layers that were higher than 5 km above mean sea level in spring, in which dust was detected more frequently at higher altitudes during the nighttime than in the daytime. This feature indicated that thinner dust layers were mainly distributed above 5 km in spring, particularly in the middle or upper troposphere [80]. From spring (MAM) to winter (DJF), the maximum occurrence frequency of dust decreased gradually, which is consistent with the region’s seasonal dust activity [83]. In summer, dust peaked at a height of 2 km above mean sea level, with values of 0.025 and 0.015 in the nighttime and daytime, respectively, then decreased sharply with height. In autumn and winter, the peak height of dust decreased to less than 1.0 km. At the same time, the occurrence frequency was also much lower than that in spring and summer. 

Generally, the seasonal and diurnal variations in the vertical distribution of polluted dust showed the same features as discussed in Section 3.2 for the Aral Sea region. Pollute dust is frequently encountered in the Aral Sea basin. The occurrence frequency of polluted dust in the daytime and nighttime, showing obvious diurnal variations at almost all levels, was mostly distributed between 0 and 6 km, peaking at around 1.9, 2.8, 1.2, and 0.8 km above the mean sea level in spring, summer, autumn, and winter, respectively, and then declining gently with height (Figure 6). The height of the peak occurrence frequency did not show any diurnal variations. In spring, the occurrence frequency in the nighttime was obviously larger than that in the daytime. In summer, the difference between the daytime and nighttime was larger than that in spring; this can be clearly seen in Figure 6b, indicating that polluted dust was more frequently observed at heights from 1 to 6 km. Polluted dust followed the same trend as in summer and was more frequently observed between heights of 0 km and 5 km in autumn, peaking at approximately 1.0 km, and both the altitude and the occurrence frequency were less than those detected in spring and summer. In winter, polluted dust was mainly concentrated at a height of less than 3 km. Compared to the vertical distribution of the dust layers at nighttime, the polluted dust layers were more easily detected in the upper troposphere due to the thinner layers. 

The polluted continental/smoke layer was mostly found from 0.5 to 2.8 km above mean sea level (Figure 7). The vertical distribution of the polluted continental/smoke layer showed the same variation feature, with significant diurnal variation in occurrence frequency; however, there was no significant diurnal variation in the height of the peak occurrence frequency. Vertically, the occurrence frequency of polluted continental/smoke in spring peaked at approximately 1.4 km above mean sea level and gradually decreased with height. In autumn, it can be clearly seen that polluted continental/smoke was more frequently observed in the nighttime compared with the daytime, and the occurrence frequency showed obvious differences. The vertical distribution of polluted continental/smoke in autumn increased slightly when compared with the distributions in spring and summer, and was largely distributed below 1.5 km in winter, at which time the occurrence frequency (larger than 0.03) was highest out of the four seasons, which can mostly be attributed to production and consumption of fossil fuel in the Aral Sea region [92].

The vertical distribution of the occurrence frequency of the clean continental layer is presented in Figure 8, showing significant seasonal and diurnal variations. Clean continental was more frequently observed in the nighttime and seldom detected in the daytime due to the thin-layer features and low occurrence frequency [80]. Therefore, observations during the nighttime are the focus of this section. In spring, clean continental was distributed below 4 km with an occurrence frequency of less than 0.01, peaking at approximately 0.5 km, and then declined gradually with increasing height. In summer, clean continental was distributed over a wide range from 0.2 to 12 km, with a value of less than 0.005. In autumn and winter, clean continental was mostly observed for heights below 2.0 km above mean sea level and peaked at 0.5 km in height, with an occurrence frequency of more than 0.02, which was much higher than that in spring and summer; this was mostly caused by human activities such as heating around the Aral Sea region. 

The elevated smoke layer was mainly distributed over a range from 2.5 to 6.0 km above the mean sea level (Figure 9). The occurrence frequency in the nighttime was much higher than that found in the daytime, which is consistent with the diurnal variation feature found in other regions [78]. In spring, the elevated smoke layer peaked at approximately 3.5 km with a maximum occurrence frequency of 0.005 in the nighttime, then decreased sharply with height. The occurrence frequency of elevated smoke in summer was less than 0.005 in the daytime and approximately 0.015 in the nighttime with significant differences, which may result from biomass burning in this season [90]. In autumn, elevated smoke showed the same features as those found in spring, peaking at 3.0 km. Both the height and the frequency of occurrence were lower than those recorded in summer. In winter, the elevated smoke layer was seldom observed in any range, and its occurrence frequency was less than 0.003 at all heights. 

### 3.4. Seasonal and Diurnal Variations of Thickness of the Aerosol Subtype Layers in the Aral Sea Region

**Figure 10:**
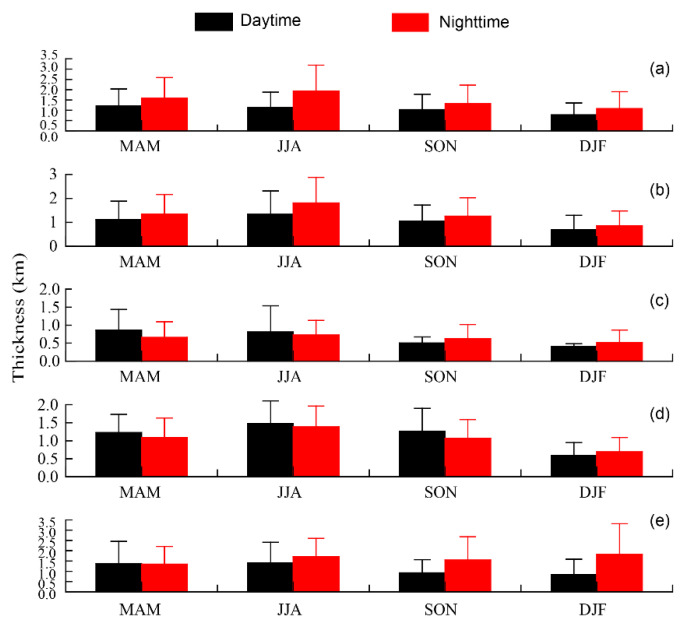
Variations in the mean thicknesses of aerosol subtype layer in the Aral Sea region in the daytime and nighttime from 2007 to 2021: (**a**) dust; (**b**) polluted dust; (**c**) clean continental; (**d**) polluted continental/smoke; (**e**) elevated smoke.

The seasonal mean thicknesses of aerosol subtype layers in the Aral Sea region in the daytime and nighttime from 2007 to 2021 are presented in Figure 10. The aerosol layer is defined in this study as the thickest continuous layer in each CALIPSO profile [63]. The seasonal thicknesses of the dust layer in the Aral Sea region were in the descending order of spring, summer, autumn, and winter, with values of 1.20, 1.14, 1.04, and 0.78 km in the daytime, respectively, while those in the nighttime were in the order of summer, spring, autumn, and winter (Figure 10a). The seasonal variation in dust layer thickness is consistent with the sand and dust activities in this region [58,83]. The dust layer in the nighttime was thicker than that in the daytime in all seasons, showing a significant diurnal difference, which mainly results from the ability of the lidar to concisely detect more dust layers at night [80]. The annual mean dust layer thicknesses were approximately 1.0 and 1.50 km in the daytime and nighttime, respectively. The same seasonal and diurnal variation characteristics of layer thickness were found in respect of the polluted dust layers. The annual mean thickness of the polluted dust was 1.05 km in the daytime and 1.31 km in the nighttime, both peaking in summer (1.33 km and 1.80 km in the daytime and nighttime, respectively) and reaching a minimum in winter (0.70 km and 0.85 km in the daytime and nighttime, respectively) (Figure 10b). The layer thickness of clean continental and polluted continental/smoke showed almost the same seasonal and diurnal variation features. The layer thickness of clean continental was the lowest among all aerosol subtypes in both the daytime and nighttime. In the daytime, the thicknesses were 0.86, 0.80, 0.50, 0.41 km in spring, summer, autumn, and winter, respectively, while in the nighttime they were 0.65 km (spring), 0.72 km (summer), 0.62 km (autumn), and 0.51 km (winter), respectively (Figure 10c). In spring and summer, the thickness in the daytime was a little higher than that in the nighttime; however, the opposite trend was observed in autumn and winter. There was no significant diurnal variation observed for clean continental due to the thinner layers [80]. The thickness of polluted continental/smoke during the daytime in summer (1.48 km) showed significant differences when compared with the number for winter (0.58 km), while the thickness in spring and autumn showed no differences (around 1.23 km). The polluted continental/smoke layer thickness in the nighttime presented the same features as in the daytime in the decreasing order of summer (1.38 km), spring (1.09 km), autumn (1.06 km), and winter (0.70 km) (Figure 10d). The layer thickness of clean continental and polluted continental/smoke showed the same seasonal variation features. The annual mean thicknesses of the elevated smoke layer were approximately 1.14 km (daytime) and 1.60 km (nighttime) over the Aral Sea region (Figure 10e). In spring and summer, the thicknesses in the daytime and nighttime did not show significant differences, while those in autumn and winter had obvious differences, presenting higher values in the nighttime than in the daytime, which may be attributed to the intense human activity such as heating in late autumn and winter. Seasonal and diurnal fluctuations in the aerosol layer thicknesses in the Aral Sea region indicated a long-standing aerosol layer over the Aral Sea region, suggesting that the region has been an active dust and salt dust source over the last two decades [93].

### 3.5. Zone Most Frequently Impacted by Aerosols in the Aral Sea Region

This section explores the zone most frequently impacted by aerosols from the Aral Sea region after presenting the variation features of the atmospheric aerosol subtypes for the Aral Sea region over the past fifteen years. The HYSPLIT model’s trajectory frequency analysis counts the number of endpoints along one trajectory that fall within each grid cell (1.0 degree in this study), then normalizes the data by the total number of trajectories [63]. The frequency analysis of air parcel trajectories provides information regarding certain regions that are regularly impacted by atmospheric aerosols from the Aral Sea region. There were three trajectory frequency classes (%) presented. The severely impacted zone has a frequency of trajectory larger than 10% but less than 100% (maximum), the moderately impacted zone has a frequency of trajectory larger than 1% but less than 10%, and the lightly impacted zone has a frequency of trajectory larger than 0.1% but less than 1% [63]. The trajectory frequency classes between 0.1% and the minimum value are not presented in the figures due to the low trajectories or lack of possibility to transport the aerosols to the downwind area of the Aral Sea.

**Figure 11:**
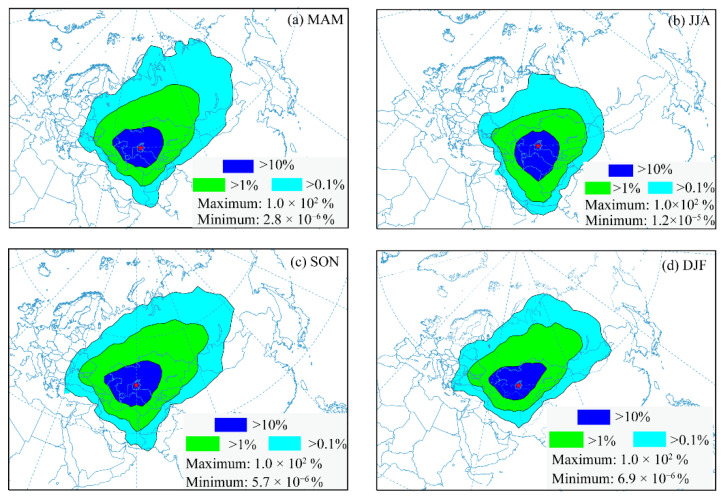
Zone most frequently impacted by aerosols from the Aral Sea region in different seasons. The red asterisk indicates the location of the Aral Sea.

**Figure 12:**
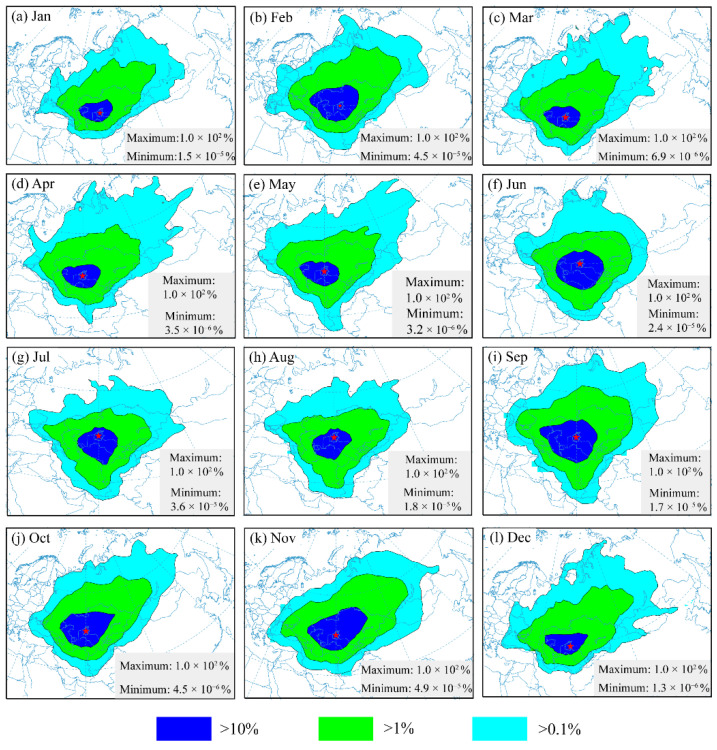
Zone frequently impacted by atmospheric aerosols from the Aral Sea region in different months. The red asterisk indicates the location of the Aral Sea.

The zone that is frequently impacted by atmospheric aerosols from the Aral Sea region showed marked seasonal features (Figure 11). In spring, the Asian high leads to prevailing northeasterly and southwesterly winds in this region [61]. The severely impacted zone (blue zone) extended westward at a low height, and was mainly distributed around the Aral Sea region, but also in neighboring areas and had the potential to cover most of Central Asia (covering an area of approximately 2 million km^2^), including western Kazakhstan and most of Uzbekistan and Turkmenistan (Figure 11a). This can be seen more clearly in Figure 12c–e. After lifting to a higher altitude, the aerosols can be transported over a long distance toward the east or northeast due to the perennial westerly belt (Figure 11a and Figure 12c–e). The moderately impacted zone (green zone) covers a larger area, including Uzbekistan, Kazakhstan, Turkmenistan, Kyrgyzstan, and part of Iran, and even the Caucasus region, extending eastwards towards Russia’s Siberia, particularly to the Tianshan Mountains, with potentially lower atmospheric aerosol concentrations (greater than 1% and lower than 10%). The lightly impacted zone (light−blue zone in the figure) in spring comprised the areas with the lowest levels of air aerosols (>0.1%), stretching to the central Siberian Plateau and eastern Siberia in the eastern branch, covering most of Russia, Mongolia and the northwest of China. 

The southward branch in spring covered the border region between Iran and Afghanistan and was more prominent and obvious in summer (Figure 11b and Figure 12f–h). The Caspian Sea and Kush–Karakoram range pressure anomalies, as well as the northern Levar wind over that region, have a considerable influence on the dusty air branch moving south along the Iran−Afghanistan border [94]. The severely impacted zone, moderately impacted zone, and lightly impacted zone all stretch toward the south, which can be attributed to the strong cyclonic activity in this region [95]. The severely impacted zone mainly covers Uzbekistan, Kazakhstan, and Turkmenistan at a lower altitude, the same as found in spring. After being raised to a high level, the aerosols are then transported toward the south for a long distance, covering most of Iran and Afghanistan and most of Kazakhstan, Uzbekistan, Turkmenistan, and Kyrgyzstan. The lightly impacted zone can be extended to the Persian Gulf and the Arabian Sea in the southerly direction and to the Tianshan Mountains region in the easterly direction, possibly affecting the vegetation and resulting in faster melting of snow and ice in mountainous areas after the aerosols settle in this region. 

The spatial pattern of the zone frequently impacted by aerosols from the Aral Sea region in autumn shows similar trends to that in spring. The zone most frequently impacted by aerosols from the Aral Sea region had nearly identical coverage and direction patterns in autumn as those found in spring (Figure 11c and Figure 12i–k). Dust activity in autumn is less active in this region [93]. At low altitudes, the high density of air parcels indicates that the severely impacted zone was transported toward the northwest (approximately 2 million km^2^), then turned to the northeast after being lifted into the middle or top of the troposphere (moderate impacted zone). When lifted into the upper troposphere, the atmospheric aerosols can be transported for a long time and over long distances because of the prevailing westerly all year round [59,61], which has the potential to transport the lower-density aerosols to positions far away from the Aral Sea region.

During winter, high pressure controls large parts of central Asia, strengthening southwesterly winds to storm magnitudes [61]. The severely impacted zone, moderately impacted zone, and lightly impacted zone all extended toward the northeast (Figure 11d and Figure 12a,b,l). High potential aerosol concentrations (severely impacted zone) were mainly located in Kazakhstan and Uzbekistan. The moderately impacted zone would cover Turkmenistan, Kyrgyzstan, and the Iranian Plateau. The aerosols were pushed toward the northeast when being elevated to a higher altitude and transported for a long duration and distance due to the persistent westerly jet stream, which extends to the Tianshan Mountains, covering Mongolia and the Siberian Plateau in the east and the Caucasus Region in the west. 

## 4. Conclusions

In this work, CALISPO and HYSPLIT were combined to gain preliminary information regarding the seasonal and diurnal variations and the most frequently impacted zone of aerosols from the Aral Sea region. The results provide new insights into the seasonal and diurnal variations of aerosol subtypes in terms of altitude profiles, layer thicknesses, and frequently impacted zones of atmospheric aerosols from the Aral Sea region. The results will also provide a reference for positioning monitoring of aeolian dust deposition, which will almost certainly be expanded and improved upon in the future.

In the Aral Sea region polluted dust and dust were the most two dominant atmospheric aerosol subtypes. The percentages of polluted dust and dust were the two highest for both nighttime and daytime, with the two accounting for over 75% of observed aerosols. 

The occurrence frequency of polluted dust, clean continental, polluted continental/smoke, and elevated smoke over the Aral Sea region showed significant seasonal and diurnal variations, while the occurrence frequency of dust only showed obvious seasonal variation. Dust and polluted dust had higher occurrence frequencies in spring and summer, while clean continental and polluted continental/smoke were more frequently observed in winter. 

The vertical distribution of aerosol subtype layers in the Aral Sea region presented significant seasonal and diurnal variations. The occurrence frequencies of all aerosol subtypes except dust showed significant diurnal variation at all levels.Dust was more frequently observed between 0.5 km and 4.3 km. Polluted dust was most commonly detected approximately between 1 and 6 km. Polluted continental/smoke layer was mainly observed in the range of 0.5 to 2.8 km, and the elevated smoke layer was mainly distributed over the range from 2.5 to 6.0 km. 

The layer thickness of dust and polluted dust showed significant seasonal and diurnal variations, and the layer thickness of clean continental and polluted continental/smoke shared the same seasonal and diurnal variation features. Seasonal and diurnal fluctuations in the layer thickness of the aerosol subtype indicated a long-standing layer of aerosols in the Aral Sea region. 

The severely impacted zone was mainly distributed around the Aral Sea region, including the western part of Kazakhstan and most of Uzbekistan and Turkmenistan, with an area of approximately 2 million km^2^. The moderately impacted zone included Kazakhstan, Uzbekistan, Turkmenistan, Kyrgyzstan, and part of Iran, as well as the Caucasus region, extending eastwards into Siberia in Russia. The lightly impacted zone stretched to the central Siberian Plateau and eastern Siberia in the east, covering most of Russia and Mongolia, as well as the northwest of China.

## Figures and Tables

**Table 1:** The mean and standard deviation (in brackets) of the percentage of aerosol subtypes in the daytime and nighttime.

	Dust	Polluted Continental/Smoke	Clean Continental	Polluted Dust	Elevated Smoke
Daytime (21,040) *	58% (8%)	9% (4%)	2% (1%)	27% (7%)	3% (2%)
Nighttime (65,964) *	25% (5%)	9% (2%)	9% (2%)	50% (4%)	6% (1%)

* This number is the total amount of CALIPSO aerosol subtype measurements.

## Data Availability

The data presented in this study are available on request from the corresponding author.
